# An HS-GC-IMS Method for the Quality Classification of Virgin Olive Oils as Screening Support for the Panel Test

**DOI:** 10.3390/foods9050657

**Published:** 2020-05-20

**Authors:** Enrico Valli, Filippo Panni, Enrico Casadei, Sara Barbieri, Chiara Cevoli, Alessandra Bendini, Diego L. García-González, Tullia Gallina Toschi

**Affiliations:** 1 Department of Agricultural and Food Science, Alma Mater Studiorum—Università di Bologna, 47521 Cesena, Italy; enrico.valli4@unibo.it (E.V.); filippo.panni2@unibo.it (F.P.); chiara.cevoli3@unibo.it (C.C.); alessandra.bendini@unibo.it (A.B.); tullia.gallinatoschi@unibo.it (T.G.T.); 2 Interdepartmental Center for Industrial Agrofood Research, Alma Mater Studiorum—Università di Bologna, 47521 Cesena, Italy; 3 Department of Pharmacy and Biotechnology, Alma Mater Studiorum—Università di Bologna, 47521 Bologna, Italy; sara.barbieri@unibo.it; 4 Instituto de la Grasa (CSIC), 41013 Sevilla, Spain; dlgarcia@ig.csic.es

**Keywords:** virgin olive oil, HS-GC-IMS, volatile compounds, chemometric analysis, sensory analysis

## Abstract

Sensory evaluation, carried out by panel tests, is essential for quality classification of virgin olive oils (VOOs), but is time consuming and costly when many samples need to be assessed; sensory evaluation could be assisted by the application of screening methods. Rapid instrumental methods based on the analysis of volatile molecules might be considered interesting to assist the panel test through fast pre-classification of samples with a known level of probability, thus increasing the efficiency of quality control. With this objective, a headspace gas chromatography-ion mobility spectrometer (HS-GC-IMS) was used to analyze 198 commercial VOOs (extra virgin, virgin and lampante) by a semi-targeted approach. Different partial least squares-discriminant analysis (PLS-DA) chemometric models were then built by data matrices composed of 15 volatile compounds, which were previously selected as markers: a first approach was proposed to classify samples according to their quality grade and a second based on the presence of sensory defects. The performance (intra-day and inter-day repeatability, linearity) of the method was evaluated. The average percentages of correctly classified samples obtained from the two models were satisfactory, namely 77% (prediction of the quality grades) and 64% (prediction of the presence of three defects) in external validation, thus demonstrating that this easy-to-use screening instrumental approach is promising to support the work carried out by panel tests.

## 1. Introduction

Thanks to their unique sensory attributes and their compositional uniqueness, extra virgin olive oils (EVOOs) and virgin olive oils (VOOs) are usually marketed at a higher price than other vegetable oils [1], frequently rendering them the object of fraudulent practices. EVOOs and VOOs can be destined for human consumption; however, lampante olive oil (LOO) is not edible and therefore not marketable. Therefore, it is very important to classify each product in the proper commercial category, and to verify that the quality degree as reported in the label corresponds to the product contained in the related recipient, in order to not mislead consumers.

Sensory analysis carried out by a specific methodology, the panel test, plays a crucial role in classification of VOOs, together with chemical-physical analytical determinations. Its main objective is to define a sample to a specific quality grade by identifying and quantifying the intensity of eventual most perceived defect and positive attribute of fruity [2,3]. The origin of positive and negative sensory characteristics in VOOs, perceived by both orthonasal and retronasal olfaction, is due to the presence of volatile molecules that depend on many factors, such as the variety of the olives and cultivation area, as well as environmental, agronomic and technological variables [4,5,6]. The qualitative-quantitative combination of six carbon atom compounds (C6) as well as five carbon atom (C5) molecules deriving from the lipoxygenase (LOX) pathway is responsible, together with others such as terpenes, for the positive notes of fruitiness and resemble characteristic secondary attributes, e.g., grass, artichoke [5]. However, in addition to these molecules, other volatile compounds may originate from fermentative and degradative microbial processes affecting sugars and proteins, as well as lipid oxidation [4]. These latter molecules have been correlated with the presence of specific negative sensory attributes and, depending on their concentration and the perceived intensity of the defect, determine a lower quality of the product, which can no longer be marketed as “extra virgin”. For this reason, the identification and quantification of volatile compounds in the aroma of VOOs are of great importance to assess its quality [7]. For this purpose, numerous analytical procedures have been adopted [8,9], among which gas chromatography (GC) is the most widely used separative technique. The combination of the results obtained from sensory and instrumental analysis can allow rapid screening of samples, increasing the number of controls and supporting sensory evaluation [10,11,12]. In recent years, alternative instrumental techniques have been developed based on different principles that emulate the responses of the human nose, tongue, and eyes [13]. In this context, HS-GC-IMS (headspace gas chromatography-ion mobility spectrometry) is an interesting screening tool. This technique is able to realize a digital fingerprint of the aroma for possible discrimination of samples in a relatively simple, rapid, and cost-effective way [14]. HS-GC-IMS was recently used in several investigations for the analysis of volatile compounds in VOOs for the determination of geographical origin [15,16] and for discrimination of quality grades [17,18,19].

In this work, a new semi-targeted analytical approach has been developed by focusing on 15 volatile compounds that were previously selected from analytical investigations within the European project Horizon 2020 OLEUM “Advanced solutions for assuring the authenticity and quality of olive oil at a global scale”, grant agreement no. 635690, and known to be associated with positive and negative sensory attributes in VOOs [8,20]. In particular, HS-GC-IMS analysis was performed on a set of 198 VOO samples and followed by development of two-category PLS-DA (partial least squares-discriminant analysis) discrimination models of which one was adopted for the first time in the classification of samples on the basis of the presence of sensory defects. Furthermore, most of the samples were evaluated by 6 different sensory panels using the decision tree developed within the OLEUM project [21]. The goal of this investigation was to establish a semi-targeted screening methodology that can support the panel test with the aim of being successfully used by olive oil companies in the future, as well as in laboratories for routine quality control analyses.

## 2. Materials and Methods

### 2.1. Virgin Olive Oil (VOO) Samples and Sensory Evaluation

A set of 198 VOO samples was analyzed. Specifically, 153 samples, collected from olive oil companies in 2018 within the European H2020 OLEUM project, were evaluated by 6 different sensory panels involved as partners in the project; based on the sensory results elaborated according to a decision tree [21], samples were classified into three quality grades according to Regulation (EU) 2019/1604: EVOO (69 samples), VOO (51 samples), and LOO (33 samples). The remaining 45 samples were evaluated sensorially by the professional committee of VOO tasters of the University of Bologna: 14 were classified as EVOO, 18 as VOO, and 13 as LOO. All samples were stored in a freezer at −18 °C until analysis, thawing them for an adequate time - until no solid phase was observable - at room temperature and shaken carefully before use. The oil recipients were kept open only for a short time and the headspace volume was always minimized.

### 2.2. Headspace Gas Chromatography-Ion Mobility Spectrometry (HS-GC-IMS): Instrumental Equipment

The analysis was performed using a GC-IMS Flavourspec^®^ instrument (G.A.S. Dortmund, Dortmund Germany) connected to a nitrogen generator for carrier/drift gas production (Microprogel, Pordenone, Italy). For injection, 100 μL of each sample headspace was withdrawn using a 2.5 mL Hamilton syringe with a 51 mm needle, through an autosampler unit, HT2000H (HTA s.r.l., Brescia, Italy), and introduced in a splitless heated injector (2 mm ID, 6.5 mm OD × 78.5 mm fused quartz glass). The analytes passed into a low polar column FS-SE-54-CB-0.5, 30 m, 0.32 mm ID, film thickness 0.5 µm (94% methyl-5% phenyl-1% vinylsilicone) for a first separation. The eluate was subjected to a second separation by IMS equipped with a tritium ionizing radioactive source at 5000 V and a 9.8 cm long drift tube (Gesellschaft für Analytische Sensorsysteme mbH, G.A.S.; Dortmund, Germany).

### 2.3. Selected Volatile Compounds

In this study, 15 volatile compounds were analyzed as two different standard mixtures (SM), coded as SMA and SMB: 3-methyl-1-butanol (purity ≥ 98.5%), propanoic acid (≥99.8%), 6-methyl-5-hepten-2-one (≥97.0%), ethyl acetate (≥99.8%), (*E*)-2-heptenal (≥95.0%), ethyl propanoate (≥99.7%), (*E,E*)-2,4-hexadienal (≥95.0%) (compounds present in the SMA) and ethanol (≥99.9%), acetic acid (≥ 99.8%), 1-octen-3-ol (≥98.0%), hexanal (≥98.0%), nonanal (≥95.0%), (*E*)-2-hexenal (≥97.0%), (*Z*)-3-hexenyl acetate (≥98.0%), 1-hexanol (≥99.9%) (compounds present in the SMB). All these reagents were supplied by Sigma-Aldrich (St. Louis, MO, USA). The aforementioned volatile standards were dissolved in fresh refined olive oil to be analyzed both individually, at a concentration of 50 mg kg^−1^, and within the two SMs (at a concentration range: 0.05–50 mg kg^−1^).

### 2.4. HS-GC-IMS Analysis of Volatile Compounds Mixtures

Mixtures of individual volatile compounds were prepared from stock solutions of pure standards prepared by dissolving each standard in fresh refined olive oil at approximately 5000 mg kg^−1^. A rapid preparation at controlled room temperature was carried out to avoid evaporation of standards. By 1:100 dilution (*w*/*w*), individual volatile compounds mixtures were prepared at about 50 mg kg^−1^, in a 20 mL headspace glass vial, weighing approximately 2 g. Next, the vial was hermetically closed with polytetrafluoroethylene septum (PTFE). The sample was incubated at 40 °C for 8 min and 100 μL of headspace was injected using a heated syringe (80 °C) into the injector (set at 80 °C). The carrier gas (nitrogen gas with inlet pressure of 4 bar) passed through the GC-IMS injector transferring the sample into the GC column, using a flow ramp set as follows: the flow was initially set at 2 mL min^−1^ (default) for 2 min, then increased to 17 mL min^−1^ for the next 8 min (70% of maximum flow) and maintained at this flow for another 20 min. Finally, the flow was reduced for the next 2 min to the predefined value (2 mL min^−1^); end of the program was set at 32 min. The analytes were separated in isothermal mode at 40 °C and introduced into the ionization chamber of the IMS where the tritium source (5000 V) ionized compounds eluting from the GC column and the ions reached the drift tube of the IMS through the shutter grid. The drift tube was maintained at a constant temperature of 45 °C. The gas flow rate of nitrogen introduced in the opposite direction of the sample into the IMS (drift gas) was 150 mL min^−1^.

In addition to being analyzed individually, the 15 volatile compounds were also determined within two different standard mixtures (SM), coded as SMA and SMB (see Section 2.3), both prepared at approximately 50 mg kg^−1^. In this way it was possible to identify each single compound in the two SMs, obtaining the advantage of processing the SMA and SMB results to evaluate the performance of the method (see Section 2.6) rather than the data of the 15 volatile compounds obtained individually, with a significant advantage in terms of time needed to perform the analysis. The 15 volatile compounds were individually identified and quantified in chromatograms.

### 2.5. HS-GC-IMS Analysis of Virgin Olive Oil Samples

We weighed 2 g of each VOO in a 20 mL headspace glass vial that was hermetically closed. Subsequently, samples were analyzed following the same method reported in Section 2.4.

For each sample, a heat map (3D chromatogram) was obtained: only the 15 selected volatile compounds were considered (see Section 2.3 and Section 2.4), thus highlighting their respective signals present in the form of a monomer and/or dimer in the chromatogram, using VOCal software (Gesellschaft für Analytische Sensorsysteme mbH, G.A.S.; Dortmund, Germany). Using a specific function of the software, it was possible to export the results to a data matrix that was used to develop the discrimination models (see Section 2.7).

### 2.6. Performance of the Method

To evaluate the performance of the method, the following parameters were taken into consideration: linearity of the 15 volatile compounds, expressed in terms of range and determination coefficient (*R*^2^); intra and inter-day repeatability, as relative standard deviation percentage (RSD%) values, calculated on the maximum intensity value of two specific volatile compounds. In this latter case, three samples, corresponding to three quality grades, were evaluated.

#### 2.6.1. Linearity

The linearity of the 15 selected volatile compounds was evaluated by developing calibration curves for each analyte built through analysis of the two standard mixtures SMA and SMB as described in Section 2.3. The starting stock solutions at approximately 10,000 mg kg^−1^ for these two mixtures were prepared by weighing each volatile standard (10 compounds for SMA and 8 for SMB) in fresh refined olive oil. For the low concentration mixture (A), the following 12 dilutions were prepared: 0.05, 0.10, 0.15, 0.20, 0.25, 0.5, 1.00, 1.50, 2.00, 2.50, 5.00, 10.00 mg kg^−1^. For the high concentration mixture (B), it was necessary to prepare 15 dilutions: 0.05, 0.10, 0.15, 0.20, 0.25, 0.5, 1.00, 1.50, 2.00, 2.50, 5.00, 10.00, 15.00, 20.00, 25.00 mg kg^−1^.

#### 2.6.2. Intra-Day and Inter-Day Repeatability

Three samples of the 198 oils (see Section 2.1) were selected to be representative for each quality grade. These were an EVOO with a median of the fruity attribute of 3.0, and a VOO and a LOO with medians of the most perceived defects of 1.7 (winey-vinegary) and 6.6 (fusty/muddy sediment), respectively. Furthermore, for each sample, two specific volatile compounds were chosen for the repeatability study: (*E*)-2-hexenal and hexanal (typical of the fruity positive attribute, in the case of hexanal when it is at low-medium concentration) for EVOO; ethanol and ethyl acetate (typical of the winey-vinegary defect) for VOO; ethyl propanoate and 3-methyl-1-butanol (typical of the fusty/muddy sediment defect) for LOO. The selection of these markers was based on the previous literature [8,20], considering the high values of their determination coefficient values (see Section 3.2.1).

Intra-day repeatability was determined based on the average RSD% values of the maximum intensity (expressed in mV), calculated on the areas of the signals related to the two volatile compounds dimers in each of the three samples selected for each quality grades, analyzing them in 7 replicates on the same day.

For inter-day repeatability, the same procedure was followed but calculating the average RSD% values on the maximum intensity of the two volatile compounds dimers in each of the three samples selected for each quality grades, analyzing them for each day for one week (7 days).

### 2.7. Data Analysis

From the HS-GC-IMS analysis, a 3D chromatogram (heat map) was obtained. Each point in the heat map is characterized by the GC retention time measured in seconds, by the IMS drift time in milliseconds, and by the intensity of the ion current signal in millivolts (mV). The raw 3D data [22] were normalized on the reactant ion peak (RIP). The RIP corresponds to the reactant ions or hydrated protons, which are generated in the ion source of the employed IMS device. The analytes interact with the RIP to generate protonated species by the displacement of water [19]. Subsequently, the maximum intensity of the areas (monomer and dimer) belonging to the 15 volatile compounds were selected and used to develop the chemometric models (normalized values). Not all 15 volatile markers had both the monomer and the dimer in the heat map. For this reason, a total of 25 signals were used rather than 30.

Principal component analysis (PCA) was used as an explorative technique to evaluate the relationships between variables and to visualize the data according to the quality grade.

Different PLS-DA models were built: a first approach was used to classify the sample according to quality grades, and a second to classify samples on the basis of the presence of defects (negative sensorial attribute). For the latter, only VOOs and LOOs were considered (115 samples, of which 49% with fusty/muddy sediment defect, 29% musty-humid-earthy and 44% rancid). PLS-DA models were developed using the PLS Toolbox for Matlab; volatile compound signals were used as variable X (mean center pretreatment), while the quality grade or presence of defects were implemented as variable Y (binary variables, 0–1).

For the quality grades, 4 classification models were built, EVOO vs. no-EVOO followed by VOO vs. LOO, and LOO vs. no-LOO following by EVOO vs. VOO; for the presence of defects, 3 models were developed based on the 3 main perceived defects in the VOO and LOO samples: musty, rancid and fusty/muddy sediment.

In all cases, the sample data set were split into a calibration/cross validation set (75% of the sample) and external validation set (25% of the sample) using the Kennard-Stone method [23]. Samples for the cross validation were selected using the venetian blind method (number of data split: 10). The threshold value useful to define the category of each sample was defined using a probabilistic approach based on Bayes’s rule.

### 2.8. Set-Up of Analytical Conditions

In order to obtain the most information in the shortest time, several analytical parameters were investigated to optimize the headspace extraction and repeatability of the analysis.

Sample conditioning: a comparison between three different settings in terms of conditioning time and temperature was carried out: (i) 40 °C/20 min, according with previous investigations dealing with a similar rapid chromatographic separation [24]; (ii) 60 °C/8 min, adopting the same conditions applied by Contreras et al. [19]; (iii) 40 °C/8 min, to take advantage of both a shorter analytical time and temperature, as in (i), more similar to the real tasting experience in the panel test procedure. Comparison of heat maps obtained from the analysis of VOO samples injected after conditioning at 40 °C/8 min and 40 °C/20 min, no differences were observed in terms of either coordinates (retention time/drift time) or intensity of the spots. For this reason, the condition 40 °C/8 min was chosen to take advantage of a temperature closer to the oral cavity (about 37 °C), through which the retro-olfactory evaluation of the VOOs takes place, and of the shorter analysis time. Using a temperature that was 20 °C higher, for the same short time (60 °C/8 min), an increase in the intensity of the spots of all the volatile compounds, both associated with positive and negative attributes, was seen. These conditions improved the sensitivity of the analysis, but a higher temperature also led to variations in the chemical-physical balance between volatile compounds of the headspace, moving away from the quali- and quantitative equilibrium occurring in the mouth. Therefore, with the aim of establishing a rapid screening procedure to support the panel test, it was decided to adopt the temperature (40 °C) that was closest to that of organoleptic evaluation, while taking advantage of the short analysis time (8 min) proposed by Contreras et al. [19].

Gas carrier flow: constant flow (isobaric analysis) and flow ramp were compared. The former has the advantage of being extremely simple even for inexperienced operators, while the second improved the separation of spots obtained in heat maps, showing better resolution. The flow ramp conditions are described in detail in Section 2.4.

GC column temperature: a comparison between 40 °C and 55 °C [19] was carried out; it was decided to adopt a temperature of 40 °C, as an evident compression of the heat map in terms of retention time was observed at 55 °C, contrasting the positive effect of the flow ramp mentioned above.

## 3. Results and Discussion

### 3.1. Selected Volatile Compounds

One of the main objectives of the H2020 EU OLEUM project is to develop instrumental methods that support the panel test [10]. Many analytical efforts have been addressed by the research institutions involved to select a list of volatile compounds, focusing on the most relevant ones, that can define sensory characteristics, both fruity and defects. Finally, 18 volatile compounds were identified as the most relevant markers: it was also decided to split these selected compounds into two mixtures (SMA and SMB), depending mainly on the presence of each one at lower or higher concentrations in VOOs. Three markers of the 18 were excluded when performing this investigation, namely octane, pentanoic acid, and (E)-2-decenal. This was due to the chemical ionization of these analytes in the IMS region that occurs if the proton affinity of the analyte is greater than that with water [25]. The alkanes, to which octane belongs, have a proton affinity less than that with water: this means that these compounds will be more difficult to ionize, consequently causing low sensitivity of the GC-IMS towards them. (E)-2-decenal was also not considered due to the low sensitivity of the instrument towards it as well as its long retention time (51 min), which is not within the working range (0–32 min); an increase of the analysis time would make this analytical approach less attractive for screening purposes. Similar considerations also apply to pentanoic acid. This semi-targeted approach also made it possible to facilitate data elaboration due to the lower amount of raw data to be processed compared to an untargeted method.

### 3.2. Performance of the Method

#### 3.2.1. Linearity

Table 1 shows that the linear range in the standard matrixes of almost all the 15 volatile compounds is narrower than the ranges discussed above. 6-methyl-5-hepten-2-one and propanoic acid showed a linear response for the entire concentration range considered for the SMA (0.05–10 mg kg^−1^). The same was observed for 1-hexanol in the SMB (0.05–25 mg kg^−1^). All other volatile compounds had smaller linear ranges; in particular, this was highlighted for ethyl acetate, ethyl propanoate, and ethanol (0.05–0.5 mg kg^−1^). This behavior should be further investigated in the future, as especially in LOOs it is well known that some of these compounds are present even at much higher concentrations [8]. Nonetheless, it should be underlined that quantification of these molecules was not one of the main objectives of this method, as it is proposed for a semi-targeted screening. Despite this, the possibility to use this instrument for quantification purposes, with the use of an internal standard and as an alternative to other techniques (e.g., SPME-GC–MS), would be interesting to investigate.

#### 3.2.2. Intra-Day and Inter-Day Repeatability

**Figure 1:**
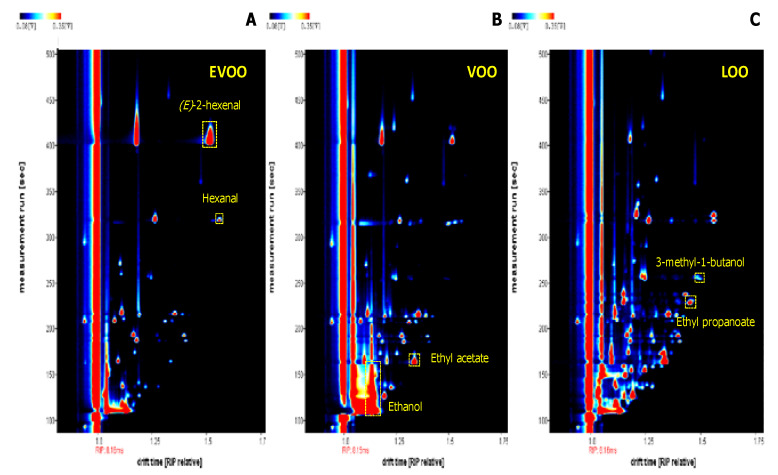
Heat maps in which the signals corresponding to the volatile compounds selected for the evaluation of intra- and inter-day repeatability have been indicated. (**A**) extra virgin olive oil (EVOO) sample with highlighted signals of (*E*)-2-hexenal and hexanal; (**B**) virgin olive oil (VOO) sample with highlighted signals of ethyl acetate and ethanol; (**C**) lampante olive oil (LOO) sample with highlighted signals of 3-methyl-1-butanol and ethyl propanoate.

Figure 1 shows the signals corresponding to the selected volatile compounds described in Section 2.6.2. The RSD% values for intra-day repeatability, calculated on the maximum intensity of the compound areas selected for the three quality grades, ranged from 1.0 to 1.7, with the only exception being hexanal, which had a higher value of 5.0. In the case of inter-day repeatability, the RSD% intervals were similar to those obtained in the intra-day experiment, with lower repeatability for ethyl propanoate (3.3) and hexanal (6.7). In any case, all these values are widely acceptable and comparable with those found in the literature [17,19,26,27]. From a recent study by Contreras et al. [28], it was observed that, working with the HS-GC-IMS in isothermal mode, the ethanol dimer signal (shown in Figure 1B) partly co-eluted with a ghost signal in the Rt and Dt dimensions [1]. For this reason, in this investigation a distinction between the ethanol signal and the ghost signal was difficult; therefore, the area considered for ethanol was given by the sum of the dimer signal plus the ghost signal.

### 3.3. Results of the Semi-Targeted Chemometric Models for the Quality Grade Classification and on the Presence of the Defects

**Figure 2:**
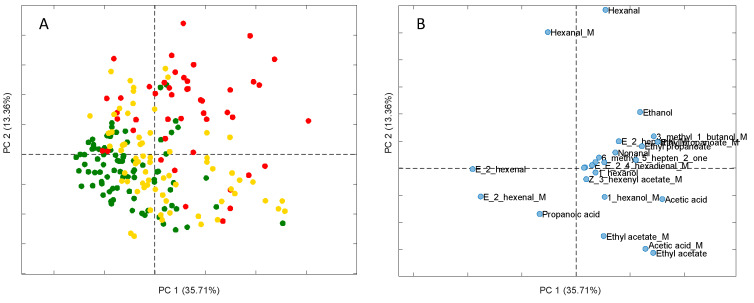
Score plot (**A**): green (EVOO), yellow (VOO), red (LOO); loading plot (**B**) obtained by principal component analysis (PCA).

The score plot of the first two PCs (35.71, and 13.36%) obtained by the PCA is shown in Figure 2A. Clear separation between the EVOO and LOO samples can be seen, while the VOOs are dispersed among the EVOOs and LOOs. The effect of the variables on each component and according to the contribution in the group separation were evaluated by a loading plot (Figure 2B). For the PC1, the greater contribution is due to the (*E*)-2-hexenal, acetic acid, 3-methyl-1-butanol and ethyl propanoate, while PC2 was strongly influenced by hexanal and ethyl acetate.

**Figure 3:**
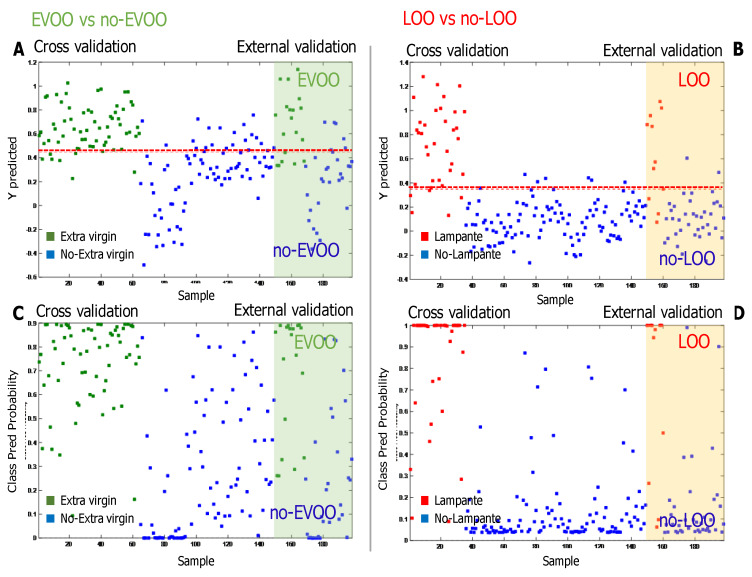
Graphical results obtained from 2 of the 4 partial least squares—discriminant analysis (PLS–DA) models for prediction of quality grade of virgin olive oils (VOOs). (**A**,**B**): values of the estimated Y variable by the model, extra virgin olive oil (EVOO) vs. no-EVOO (**A**) and lampante olive oil (LOO) vs. no-LOO (**B**), in cross and external validation. (**C**,**D**): values of the class prediction probability by the model, EVOO vs. no-EVOO (**C**) and LOO vs. no-LOO (**D**), in cross and external validation.

Concerning the PLS–DA results, the values of the estimated Y variable (quality grades) obtained by the model in cross and external validation are shown in Figure 3A,B. The dotted line identifies the threshold value used to define the categorization of samples to different classes. In particular, the examples of two PLS–DA models are shown: Figure 2A represents the EVOO vs. no-EVOO model, while Figure 2B shows the LOO vs. no-LOO model.

The results, in terms of percentage of correctly classified samples, are reported in Table 2; the percentages ranged from 67% to 95%. Considering the external validation data, the best result in terms of prediction was obtained for the LOO vs. no-LOO model (95%), while the worst was the EVOO vs. VOO model (67%). This is likely due to the fact that some of the VOO samples could be considered as borderline compared to EVOOs since they have similar profile patterns of volatile compounds, and are more difficult to be discriminated by the EVOO vs. VOO model. The results are comparable with those found in similar studies [11,19]. In the targeted approach by Contreras et al. 2019 [19], the results, in terms of prediction obtained by the models, are in agreement with those reported herein. In particular, the highest percentages of correctly classified samples are obtained for the LOO vs. no-LOO model. Similar results (84% of samples correctly classified, calculated as mean % among the three commercial categories) have also been obtained from PLS-DA models based on the SPME-GC-MS analysis, as in the study by Quintanilla-Casas et al. 2020 [11] where an EVOO vs. no-EVOO followed by VOO vs. LOO approach was applied.

**Figure 4:**
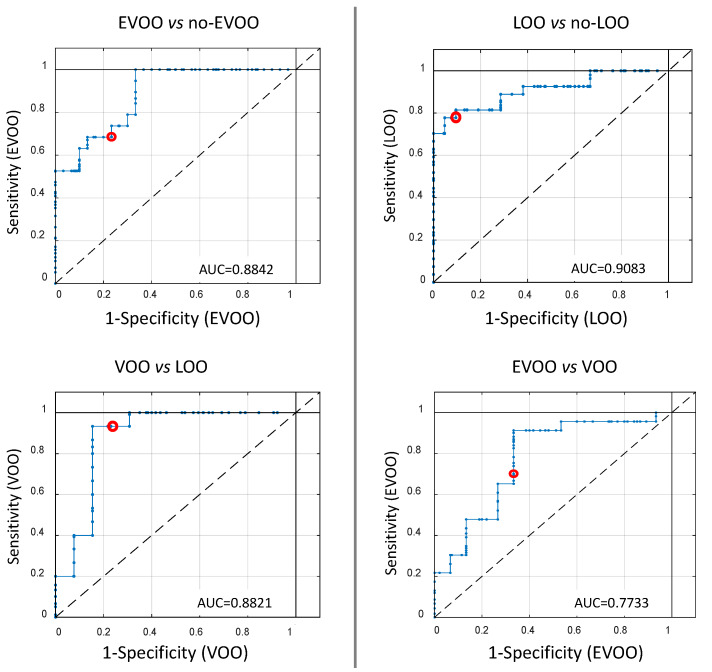
Receiver operating characteristic (ROC) curves of PLS-DA models used to discriminate samples according to quality grade. The red circle identifies selected sensitivity and 1-specificity values for the prediction model.

For all PLS-DA models, sensitivity (number of samples predicted as in the class divided by number actually in the class) and specificity (number of samples predicted as not in the class divided by actual number not in the class) were evaluated by receiver operating characteristic (ROC) curves (Figure 4). For each model, the sensitivity and 1-specificity are marked by a red circle. The area under the curve (AUC) identifies the degree of discrimination. The best discrimination was achieved for the LOO vs. no-LOO model (AUC = 0.9083), while the worst was observed for the EVOO vs. VOO model (AUC = 0.7733) as confirmed by the classification percentage.

**Figure 5:**
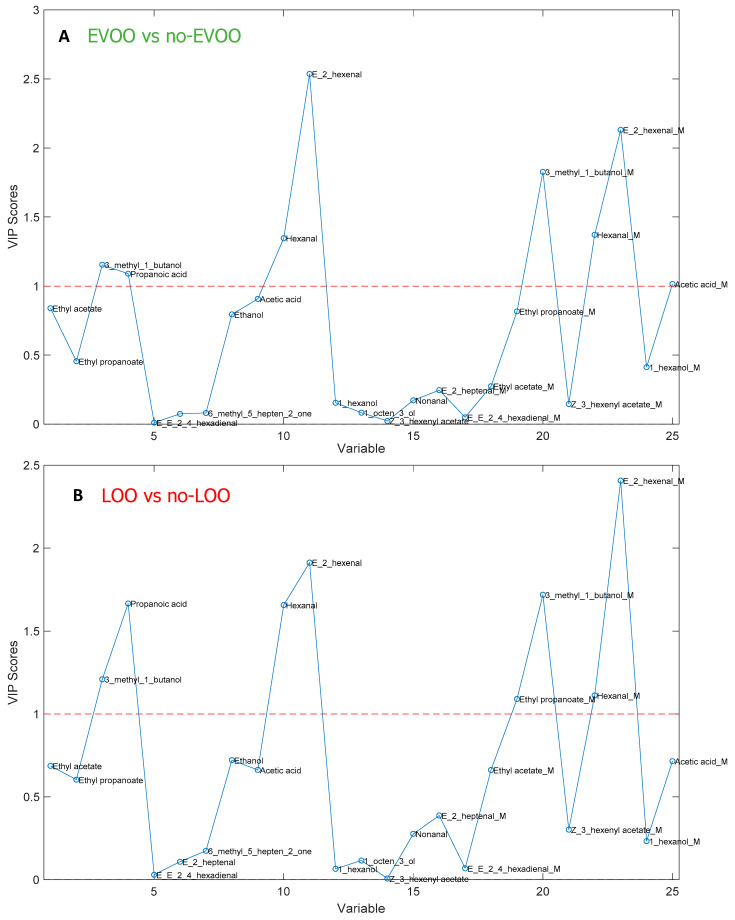
(**A**) Variable importance in projection (VIP) score obtained by the EVOO vs. no-EVOO model. (**B**) Variable importance in projection (VIP) score obtained by the LOO vs. no-LOO model.

The VIP (variable importance in projection) score obtained by the PLS-DA models shows that the volatile compounds with the highest contribution to sample discrimination, as shown in Figure 5, are (E)-2-hexenal and hexanal for EVOOs, while they also include 3-methyl-1-butanol, ethyl propanoate, and propanoic acid for LOOs, in agreement with those evaluated by PCA. In reality, these molecules are well-known markers associated with the fruity attribute or with sensory defects [8,20].

The results in terms of probability in belonging to the different categories are shown in Figure 3C,D. Figure 3C refers to the category EVOO, while Figure 3D refers to category LOO: the higher a sample is placed in the graph, the higher the probability for which it is classified accordingly to quality grade. As a consequence, samples classified as no-EVOO for Figure 3C and no-LOO for Figure 3D are located in the bottom area of the graph. In Figure 3C, it can be seen that 63% of EVOO samples and 70% of no-EVOO are classified with a probability higher than 70%. For the LOO and no-LOO samples, the corresponding percentages were 63% and 87%, respectively (Figure 3D).

The percentage values of correctly classified samples, obtained from the PLS-DA models based on the presence of 3 sensory defects (musty, rancid, fusty/muddy sediment), are shown in Table 3. The percentages ranged from 48% to 80%. The best result was obtained for the musty vs. no-musty model, even if the percentages (both in cross and external validation) for this model are not entirely satisfactory. The prediction of the presence/absence of a defect in VOO samples is very challenging. The complexity is also due to the fact that each defective sample analyzed was often characterized by more than one defect, as commonly occurs in VOOs. Future studies will aim to improve this issue by analyzing a greater number of defective samples.

## 4. Conclusions

The panel test is fundamental to discriminate the quality grade of EVOOs and to distinguish them from the virgin and lampante categories, which is relevant since the latter is not edible and must be subjected to refining.

This sensory analysis is strategic during both blending and bottling of VOOs and EVOOs carried out by olive oil companies, and within the quality control performed by official bodies. In all these cases, thousands of samples must be evaluated sensorially over the course of a year. To speed up this bottleneck, the proposed HS-GC-IMS method consists in a screening to pre-classify samples, before the panel test, into different clusters: (a) those with a probability of belonging to a commercial category greater than an established threshold (to be defined by each olive oil company, laboratory, or other user); (b) others (not reaching this threshold) that must be treated as insufficiently robustly classified. For the former, the execution of the panel test is less urgent than for the latter. In both cases, the result obtained in terms of prediction must be confirmed—or disconfirmed—by the panel test outcomes, which has legal value. An alternative or complementary use of the prediction result, in terms of confirmation or disconfirmation, can be in case of discordant classifications by different panels, where it can work as an additional information.

The promising models developed herein to predict the quality grade and presence of three sensory defects (musty, rancid, fusty/muddy sediment) provided percentages of correctly classified samples in external validation from 67% to 95%, for the quality grade prediction model, and from 48% to 80%, for the presence of each of the aforementioned defects.

Moreover, the method showed good results in terms of linearity and intra- and inter-day repeatability, although additional investigations are needed before it can be implemented commercially; furthermore, to test the performance of this approach, inter-laboratory tests involving independent laboratories will be carried out in the future.

For routine quality control, we suggest dividing the classification in two phases, firstly clustering LOO vs. no-LOO to identify non-edible samples (LOO) before being assessed by panelists, and then classifying EVOO vs. VOO. The reliability of the model can be improved upon by increasing the number of the samples to be included in the calibration, as long as they are robustly classified sensorially, e.g., by more panels with a decision tree, such as in the present paper. Furthermore, to establish its own predictive model, each laboratory could also select an internal threshold probability to discriminate between samples with acceptable and uncertain classification, and integrate this analytical information into their respective traceability systems. The possibility to use a common prediction model in different laboratories, using the same analytical conditions, can also be explored in the future, depending on the reproducibility of the signals (to be evaluated in the upcoming inter-laboratory tests) and, secondly, given the effective availability and willingness of each laboratory to share their data with others. A calibration data sharing, e.g., in a databank that could be effectively used by official control bodies or to favor harmonization and proficiency of countries that apply the same standards to olive oil.

## Figures and Tables

**Table 1:** Parameters considered for evaluation of the linearity of the volatile compounds in standard mixtures SMA (from compound 1 to compound 7) and SMB (from compound 8 to compound 15). The compounds are arranged by retention time in the respective SMA and SMB.

Volatile Compounds	Rt ^a^ (s)	Dt ^b^ (ms)	Calibration Curve Equation	Linearity Range (mg kg^−1^)	(R^2^) ^c^
1. Ethyl acetate	170	10.908	y = 672.5x + 70.5	0.05–0.5	0.980
2. Ethyl propanoate	230	11.844	y = 549.7x + 9.6	0.05–0.5	0.978
3. Propanoic acid	218	9.102	y = 15.3x + 68.4	0.05–10	0.932
4. 3-methyl-1-butanol	259	12.203	y = 279.9x + 43.6	0.05–1.5	0.986
5. (*E,E*)-2,4-hexadienal	522	11.827	y = 87.3x + 27.8	1.5–10	0.982
6. (*E*)-2-heptenal	639	13.71	y = 18.4x + 175.6	1.5–10	0.969
7. 6-methyl-5-hepten-2-one	749	9.588	y = 72.2x + 162.5	0.05–10	0.994
8. Ethanol	121	9.255	y = 345.4x + 150.4	0.05–0.5	0.980
9. Acetic acid	149	9.434	y = 14.5x + 42.7	0.10–25	0.982
10. Hexanal	317	12.723	y = 198.3x + 23.3	0.05–1.5	0.991
11. (*E*)-2-hexenal	404	12.358	y = 47.3x + 7.3	0.10–10	0.989
12. 1-hexanol	450	13.415	y = 32.9x + 83.8	0.05–25	0.988
13. 1-octen-3-ol	733	9.451	y = 33.0x + 176.2	0.05–20	0.996
14. (*Z*)-3-hexenyl acetate	846	14.908	y = 6.9x + 281.7	5.0–25	0.989
15. Nonanal	1554	12.128	y = 5.1x + 138.0	0.05–15	0.990

^a^ retention time; ^b^ drift time; ^c^ determination coefficient.

**Table 2:** Percentages of correctly classified samples by the 4 PLS–DA models for the quality grade classification of VOOs (EVOO vs. no-EVOO; LOO vs. no-LOO; VOO vs. LOO; EVOO vs. VOO).

Category	Calibration	Cross Validation	External Validation
EVOO	91%	89%	74%
no-EVOO	84%	75%	77%
LOO	89%	86%	73%
no-LOO	94%	94%	95%
VOO	92%	91%	87%
LOO	83%	76%	77%
EVOO	74%	73%	70%
VOO	80%	80%	67%

**Table 3:** Percentages of correctly classified samples by the 3 PLS–DA models to determine the presence of defects in virgin olive oils (musty vs. no-musty; rancid vs. no-rancid; fusty/muddy sediment vs. no-fusty/muddy sediment).

Defects	Calibration	Cross Validation	External Validation
Musty	71%	63%	60%
No-musty	81%	80%	80%
Rancid	81%	78%	62%
No-rancid	69%	64%	64%
Fusty/muddy sediment	82%	79%	67%
No-fusty/muddy sediment	67%	58%	48%
